# Decarburization in Laser Surface Hardening of AISI 420 Martensitic Stainless Steel

**DOI:** 10.3390/ma16030939

**Published:** 2023-01-19

**Authors:** Aprilia Aprilia, Niroj Maharjan, Wei Zhou

**Affiliations:** 1 School of Mechanical and Aerospace Engineering, Nanyang Technological University, 50 Nanyang Avenue, Singapore 639798, Singapore; 2 Advanced Remanufacturing and Technology Centre (ARTC), Agency for Science, Technology and Research (A*STAR), 3 CleanTech Loop, Singapore 637143, Singapore

**Keywords:** decarburization, carbon loss, laser hardening, furnace heat treatment, stainless steel, simulation

## Abstract

Decarburization deteriorates the surface mechanical properties of steel. It refers to the loss of carbon from steel’s surface when exposed to an open-air environment in elevated-temperature conditions. Despite the short interaction time and fast thermal cycle of the laser surface-hardening process, decarburization may still occur. This paper investigates if decarburization occurs during the laser surface hardening of AISI 420 martensitic stainless steel. For comparison, surface-hardening results and decarburizations in a conventional air furnace-heated hardening process (water-quenched and air-cooled) of the same steel material were also investigated. Decarburization seems to have occurred in the laser surface hardening of AISI 420SS. However, the decarburization might not be significant, as the hardness of the steel’s surface was increased more than three times to 675 HV during the laser surface hardening, and the hardness drop due to decarburization was estimated to be only 3% with the decarburization depth of 40 μm. Simulations using ThermoCalc software to get the carbon concentration profiles along the depth for both laser-hardened and furnace-heated samples were also investigated.

## 1. Introduction

Decarburization is a surface degradation phenomenon that involves the removal of carbon at the near top surface area of a steel during exposure to air at an elevated temperature and defined time as temperature–time-related processes [1]. This loss can largely influence the material properties of the material surfaces, such as lower strength, lower wear resistance, and lower fatigue resistance [2,3]. Decarburization remains a persistent problem in high-temperature heat treatment processes in industrial operations such as forging and rolling. Any process of high-temperature treatment of steel without a protective atmosphere can lead to decarburization [4].

Laser surface hardening is a surface modification method which induces a laser beam onto the material surfaces to improve its surface hardness and wear resistance [5]. Unlike the conventional heat treatment, laser surface hardening generates martensitic phase transformation on the targeted surface through rapid heating and cooling without affecting its bulk material. This method is widely used in automotive and heavy machinery industries in increasing the wear resistance of gears, bearings, and shafts [6]. Despite the short interaction time in the rapid heating kinetics of this process, there is still a possibility of carbon loss at the near top surface of the induced area. Carbon at a high temperature is more active with oxygen atoms compared to with iron atoms. The decarburization reaction between [C] and [O] would occur in the near-surface region producing CO gas. Some studies have reported the possibility of decarburization after laser surface treatment as one of the reasons for the degraded surface mechanical properties. Yan et al. [7,8] investigated the surface mechanical properties of plasma nitride and laser-quenched steel specimens and they found that there was a sharp decrease in the surface hardness, to which they attributed decarburization as one of the causes.

Our previous study investigated the decarburization of plain carbon steel AISI 1055 steel and 50CrMo4 alloy steel during the laser surface-hardening process [9]. We found that comparing to the plain carbon steel, there is almost no carbon loss from the surface of 50CrMo4 alloy steel during the laser surface-hardening process. This is attributed to the presence of alloying elements in the alloy steel, that may have retarded the carbon mobility in the austenite. In this study, we extend the process to another widely used alloy steel, AISI 420 martensitic stainless steel (AISI 420SS). AISI 420SS is a widely used material in aerospace, valve, pump and petroleum machinery industries due to its high corrosion resistance, shock resistance, and high plasticity properties [10,11,12]. However, it can have low hardness and poor wear resistance properties, depending on the heat treatment and delivered state, which limits its applications in some industries [13,14,15]. Laser surface hardening could be a useful surface modification method to improve the surface hardness and wear resistance of AISI 420SS. This study investigates whether decarburization occurs during the laser surface hardening of AISI 420SS. Decarburization is not desirable as it could degrade the steel’s surface mechanical properties. For comparison purposes, surface-hardening results and decarburizations of conventionally air furnace-heated sample (water-quenched and air-cooled) will also be investigated.

## 2. Experimental Procedure

### 2.1. Materials

**Figure 1:**
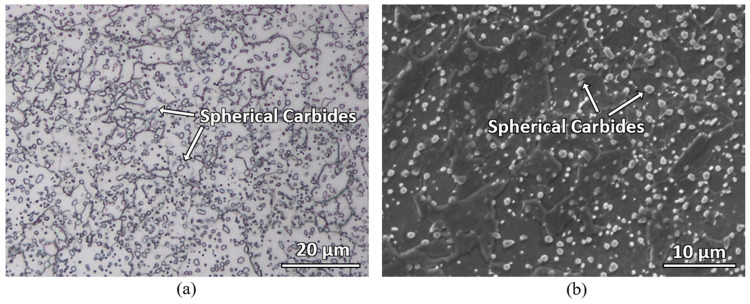
(**a**) Optical micrograph and (**b**) secondary electron micrograph of the as-received AISI 420 martensitic stainless steel showing spherical carbides distributed randomly in the matrix.

The material used in this study is an AISI 420 martensitic stainless-steel plate, whose chemical composition, as provided by the manufacturer, is shown in Table 1. Specimens with the dimension of 40 mm × 40 mm × 10 mm were subjected to a vacuum stress relief heat treatment at 650 °C for 2 h. The specimen was etched with Kalling’s No 2 etchant and an annealed microstructure with large number of spherical carbides randomly distributed in the matrix was observed (see Figure 1). The average microhardness of this material is 178 ± 5 HV. Laser surface hardening was carried out on the top surface of the specimen and characterization was carried out on the cross-sectional plane of the laser tracks.

### 2.2. Laser Surface Hardening

**Figure 2:**
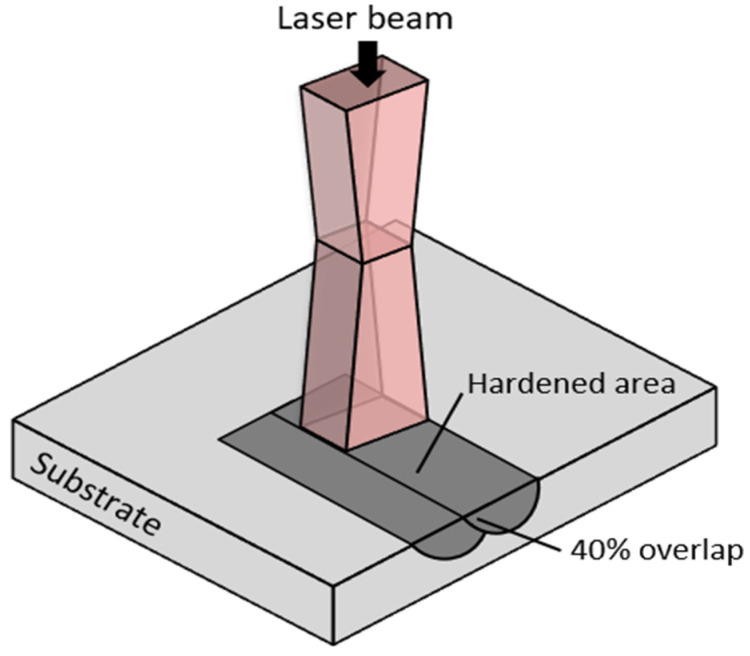
Schematic of the laser surface hardening process with 40% overlapped scanning.

Laser surface hardening was performed using LaserTec 65 3D system (DMG Mori, Bielefeld, Germany), which uses a high-power diode laser of 1064 nm wavelength. The laser beam was focused through a focusing lens producing a beam spot diameter of 3 mm at the specimen surface with a top-hat profile beam at a working distance of 13 mm. Different operating parameters were tried to determine the optimal parameters to achieve a hardened surface without any substantial surface melting. Based on the analysis, a laser power of 480 W and a traverse speed of 20 mm/s was chosen, which delivered an energy density of about 10.19 J/mm^2^ at the surface. The beam spot was scanned along the surface to perform single pass laser hardening with an overlap of 40% (see Figure 2). The experiment was carried out at room temperature, ambient pressure, and air environment.

### 2.3. Furnace-Heated Hardening

To compare and understand the surface hardening result and the decarburization in AISI 420 martensitic stainless steel, conventional furnace-heated hardening of AISI 420SS was also carried out. The sample was heated in an air box furnace at 980 °C for 2 h and subsequently quenched in a water tank for rapid cooling. The temperature of 980 °C was set, as this is within the common hardening temperature range for AISI 420 martensitic stainless steel. Another furnace-heated sample that was naturally cooled to the room temperature in an open-air environment was also carried out for comparison.

### 2.4. Characterizations

The top surface of the laser-hardened area was characterized using a 3D laser scanning confocal microscope (Keyence VK-X260K, Osaka, Japan) with a z-pitch scanning of 1.0 μm. Afterward, both furnace and laser-hardened samples’ cross-sections were mechanically ground and polished using standard metallographic sample preparation techniques. A 0.25 μm colloidal silica suspension was used in the final polishing step, and the specimens were then etched using Kalling’s No 2 reagent (5 g CuCl_2_, 100 mL HCl and 100 mL ethanol) for 10 s. Stereo micrographs were obtained using a stereo microscope Olympus SZX7 (Olympus, Tokyo, Japan), whereas the optical micrographs were obtained using light optical microscope Zeiss Axioskop 2 MAT (Carl Zeiss AG, Oberkochen, Germany). Secondary electron micrographs were obtained using scanning electron microscope JOEL 5600LV SEM (JOEL, Tokyo, Japan) with the accelerating voltage of 15 kV. Backscattered electron micrographs and electron backscattered diffraction (EBSD) maps were also obtained using JSM-7600F (JOEL, Tokyo, Japan) field emission scanning electron microscope mounted with an Oxford Instruments detector with an accelerating voltage of 20kV and step size of 0.5 μm (0.2 μm for the laser-hardened region and 0.3 μm for the furnace hardened region).

In addition to the microstructural characterization, hardness measurement characterization was also carried out to investigate the decarburization phenomenon. ASTM E1077-14 was used as the guideline [16]. The decarburization is deemed to have occurred if there is a significant difference in hardness observed at the near top surface of the material compared to the bulk carbon location hardness. The total decarburization depth is defined as the distance from the material’s top surface to the location where the bulk carbon (hardness) is found.

Microhardness tests were carried out on the sample cross sections using Vickers microhardness indenter with 300 gf at 10 s dwell time for the furnace-heated sample, and 100 gf at 15 s dwell time for the laser-hardened sample. A depth interval of 200 μm was used for the furnace-heated sample, whereas a 50 μm depth interval was used for the laser-hardened sample. Five microhardness data were collected at every depth location, and the average microhardness and standard deviations were calculated.

Berkovich nano hardness indentation was also used at the very near top surface of the laser-hardened sample to obtain a higher depth resolution result in better quantifying surface decarburization. Nanoindentations were made using Agilent G200 (Agilent Technologies, CA, USA) nano indenter machine with a Berkovich indenter. A constant depth limit of 2000 nm and surface approach velocity of 10 mm/s were used to create a series of indents with the depth interval of 20 μm on the laser-treated surface. For each depth locations, ten nano hardness data were collected to obtain the average nano hardness and standard deviation. The hardness of each point was measured using the depth range of 1000–1800 nm, where the hardness is more stable.

## 3. Results and Discussion

### 3.1. Laser-Hardened Sample Characterization

**Figure 3:**
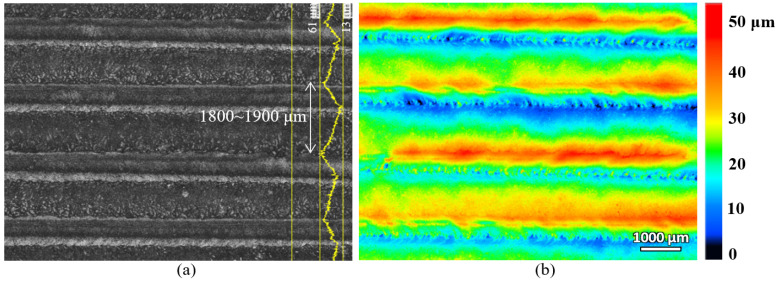
The surface profile of the laser-hardened sample showing multiple parallel tracks consisting of peaks (red) and valleys (blue): (**a**) laser intensity image (**b**) surface height image.

The laser-hardened sample’s top surface was scanned with a laser confocal microscope and the scanning result is shown in Figure 3. As seen from the figure, the top surface profile, after the laser hardening process, is made of few overlapping parallel tracks of the melted surface that form several peaks (red colour in the height colourmap) and valleys (blue colour in the height colourmap) on the surface. The peak-to-peak distance is about 1800 to 1900 μm, and the vertical distance of peak-to-valley is about 48 μm. The laser beam spot diameter used for this laser surface-hardening process was 3 mm, and the overlapping of the track scanning was set at 40%. The surface roughness of the laser track was also measured, and it was about 0.8 μm, which does not show a significant difference from the pre-hardened surface (about 0.7 μm).

**Figure 4:**
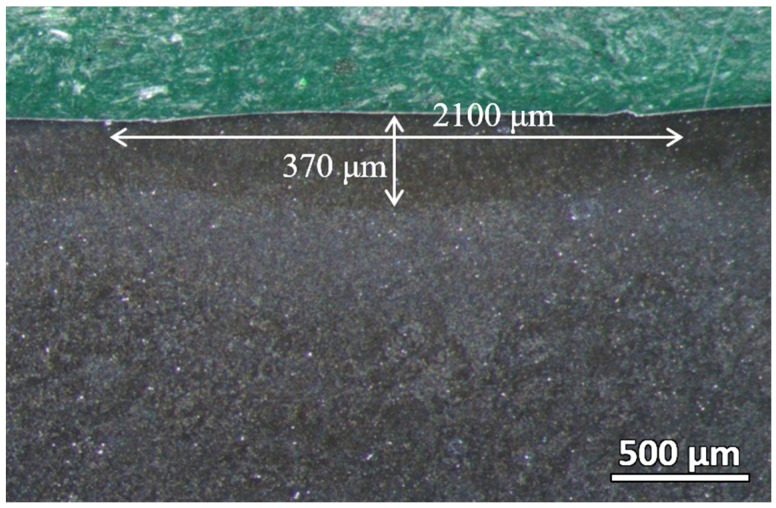
Melt pool dimension of the laser-hardened sample viewed by stereo microscope.

The cross-section of the laser-hardened sample was then polished and viewed under a stereo microscope. As shown in Figure 4, the laser-hardened sample’s melt pool, where the full melting occurs, can be clearly distinguished from the base material. The depth of this melt pool is about 370 μm, and the width for each track of the melt pool is about 2100 μm, with a slight overlapping between two adjacent tracks. The melt pool geometry is highly dependent on the process’s heat input, which is correlated with the laser power and scanning speed. By increasing the laser power or decreasing the scanning speed, the penetration depth of the hardening process can be increased.

**Figure 5:**
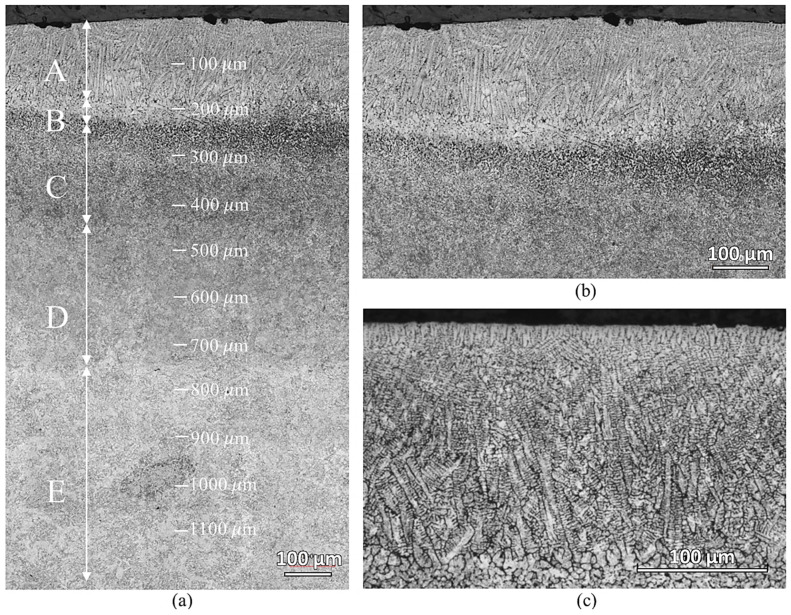
Optical micrographs of the laser-hardened sample: (**a**) low magnification micrograph showing five distinct microstructure zones; (**b**,**c**) higher magnification micrographs showing the microstructure of laser-hardened region.

Figure 5 shows the microstructure of the laser-hardened sample at various depths from the top surface. There are five distinct microstructure zones observed, which are marked with capital letters from A to E. In Zone A, down to a depth of about 200 μm, oriented dendrites appear—showing total surface melting has occurred. Another partially molten zone, zone B, about 50 μm thick, is observed beneath the completely molten layer. Zone C and D are both the transformation-affected zones but have different affected degrees. Zone E is where the material looked as in its initial condition. The dendrites in zone A were caused by the ferrite segregation at high temperature and the rapid solidification of the material during the process [17]. The interdendritic space consists of martensite microstructure. This martensite was formed during rapid cooling from carbon-enriched austenite. During the laser hardening process, the surface regions are heated to a temperature much above the usual austenitization temperature for AISI 420, resulting in non-isothermal austenitization. As the surrounding material acts as an efficient heat sink, heat is transported away from the surface region through thermal conduction, inducing rapid cooling. Austenitized material, provided with sufficient carbon quantities, forms martensite upon the quenching. The presence of martensite in zone A results in a hard and wear-resistant surface of the laser-hardened material.

**Figure 6:**
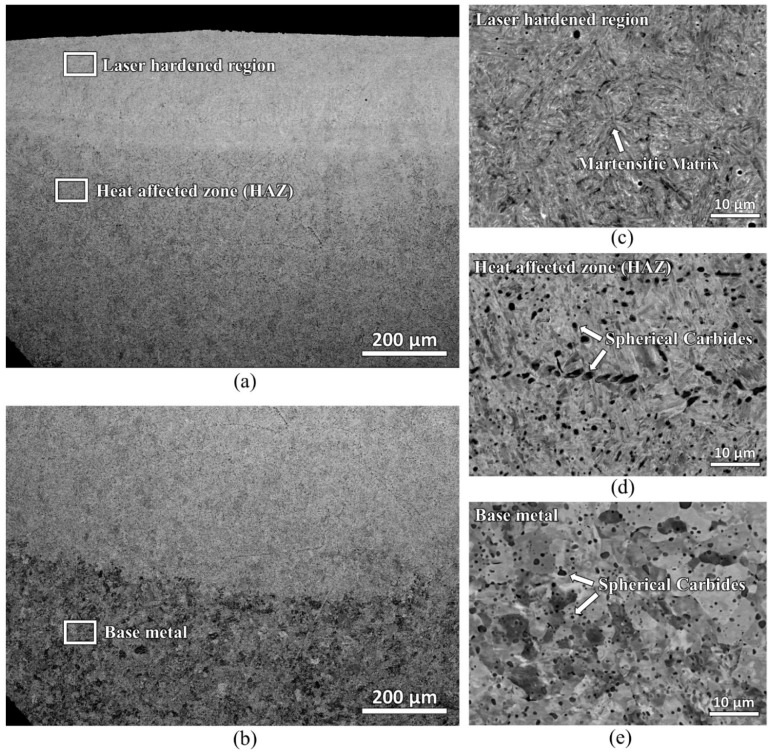
Backscattered electron micrographs of laser-hardened sample showing the microstructures of three different locations: laser-hardened region, heat-affected zone (HAZ) and base metal: (**a**,**b**) lower magnification; (**c**–**e**) higher magnification.

The cross-section of the laser-hardened sample was also viewed in a scanning electron microscope and studied with an electron backscatter diffraction detector. Figure 6 shows the backscattered electron micrographs of the laser-hardened sample. A microstructure with uniformly distributed spherical carbides in the ferrite matrix was found at the base material. In the heat-affected zone, locally coarser carbides are detected; they can result from potential coarsening (Ostwald ripening) if time and temperature are beneficial. Lastly, in the laser-hardened region, most carbides were found to have already dissolved with martensitic matrix remaining.

**Figure 7:**
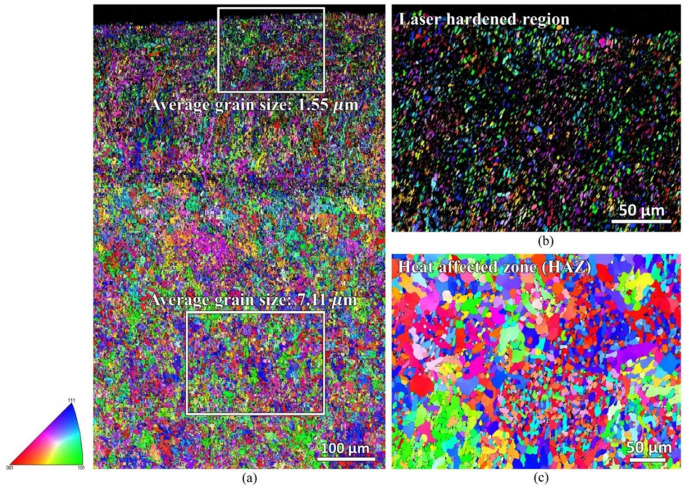
EBSD IPF-Y map showing different grain orientations and size of the laser-hardened sample: (**a**) low magnification; (**b**,**c**) high magnification of laser-hardened and heat affected zone (HAZ) regions.

Figure 7 shows the EBSD inverse pole figure (IPF-Y) map of the laser-hardened sample, which shows a different grain orientation of the steel. During the EBSD process, difficulty in indexing was faced at the near top surface area of the sample, resulting in a significant zero solution. This indexing difficulty might be due to the significant high compressive residual stress induced during the laser-hardening process. A residual stress measurement using Pulstec μ360 machine that employs cos α method was carried out. Instrument specifications with tube voltage of 30 kV, current of 1 mA, X-ray source (Cr Kα/Ni Kβ filter), and X-ray beam (wavelength = 2.29 Å, energy = 5.4 keV) were used. The residual stress measured at the near top surface is −295 MPa for the longitudinal direction and -468 MPa for the transverse direction. Another noticeable observation from the EBSD IPF mapping is the different grain sizes of the sample at two different zones: the laser-hardened zone and the heat-affected zone. The average martensitic grain size of the laser-hardened zone was measured to be of about 1.6 μm, whereas, at the heat-affected zone, its ferrite grain size is of about 7.1 μm. The original ferrite grain size of the as-received AISI 420 plate was about 6.4 μm.

**Figure 8:**
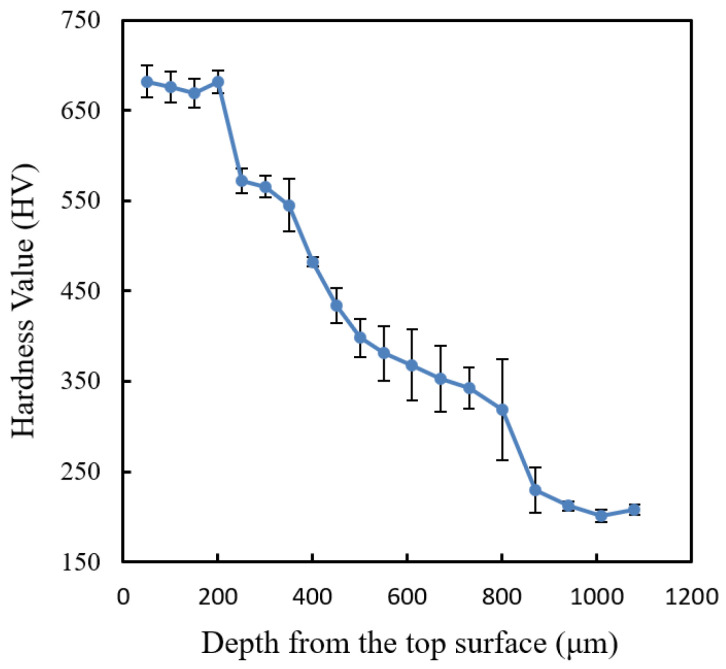
Vickers microhardness profile showing a decrease of microhardness along with the depth down to 900 μm from the top surface.

As seen from the micrographs, the occurrence of surface decarburization could not be clearly defined and quantified due to the martensitic transformation in the laser-hardened region. Therefore, hardness measurement to quantify the surface decarburization of the sample was used instead. Hardness measurement may serve as a good indicator of carbon removal during the surface laser treatment, as the hardness of martensite in steel is usually directly related to its carbon content [18]. However, it is to be noted that this hardness determination of decarburization will serve as an indication only. The changes in hardness may also be affected by other factors, such as grain growth, dislocation density variation, secondary carbides variations, etc. Therefore, a further verification on the decarburization using simulation on the carbon content changes after heat treatment processes will also be carried out in the next section. Figure 8 shows the hardness profile of the laser-hardened AISI 420SS sample measured using Vickers microhardness.

**Figure 9:**
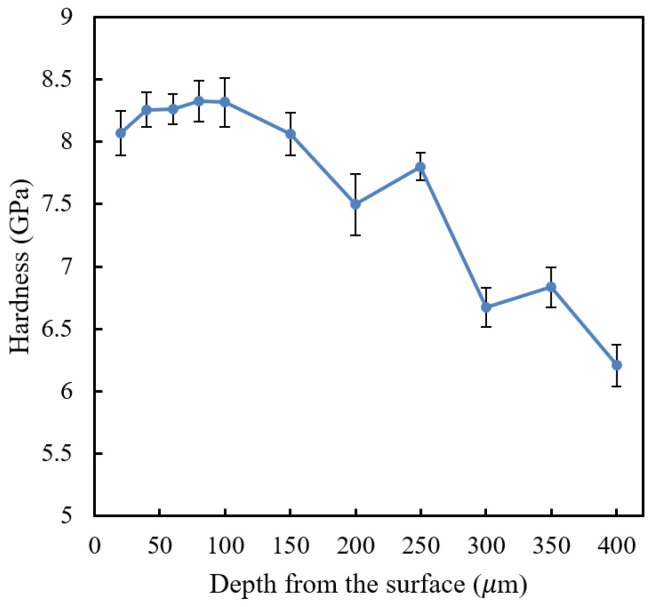
Berkovich nano hardness profile showing a slight decrease in hardness near the top surface of the laser-hardened 420SS.

As seen from the Vickers hardness profile in Figure 8, the laser-hardened region has the microhardness of about 675 HV, whereas the substrate has the microhardness of about 200 HV. This shows that laser surface hardening increases the hardness of AISI420 SS material by more than three times. For the decarburization observation, according to this microhardness result, surface decarburization was observed not to have occurred in the sample. There is no decrease in microhardness near the top surface of the sample. However, this observation is only valid for surface decarburization with the depth of more than 50 μm. Surface decarburization with the depth of less than 50 μm could not be detected as the microhardness measurement resolution was only about 50 μm. For this reason, another hardness measurement using Berkovich nano indentation machine was carried out near the top surface of the sample with a smaller measurement interval of 20 μm. Figure 9 shows the nano hardness profile of the laser-hardened AISI 420SS sample measured using Berkovich nano hardness.

As seen from nano hardness profile result in Figure 9, there is a decrease in nano hardness near the top surface of the sample. The first depth location of the measurement (20 μm from top surface) has a notably lower nano hardness compared to that of the subsequent points. This shows that decarburization might have occurred in the laser surface hardening of the AISI 420SS; however, it may not be substantial. The hardness drop was estimated to be only about 3% with the decarburization depth of about 40 μm. 

The hardness measured through Vickers microhardness was in HV unit, whereas the Berkovich nano hardness was in GPa unit. Briefly, 1 GPa is approximately equivalent to 101.968 HV. Figure 9 shows the nano hardness profile measured. Comparing it to the Vickers microhardness results of the same depth location, the Berkovich nano hardness result is found to be relatively higher. This could be due to several reasons. Firstly, it is due to the indentation size effect. The hardness value decreases with the increasing indentation load. The load used in the Vickers microhardness measurement is higher than the load used in the Berkovich nano hardness measurement. Secondly, the nanoindentation analysis uses projected contact area at peak load A_c_ instead of residual projected area A_r_ used in microhardness analysis [19]. A_c_ is always smaller than A_r_; therefore, the measured nano hardness would be larger than the measured microhardness. Thirdly, in nanoindentation, the contact area is more localized, and it is usually within a single grain. In contrast, with Vickers microhardness indentation, the contact area is wider and may include some dislocations (e.g., grain boundary), which would result in a different hardness value. It is also to be noted that conversion of hardness between different methods is not mathematically exact [20]. Different indenters, loads and material homogeneity at different scales complicate the problem. Therefore, in this study, the nano hardness unit is kept at its measured unit, GPa.

### 3.2. Furnace-Heated Sample Characterization

**Figure 10:**
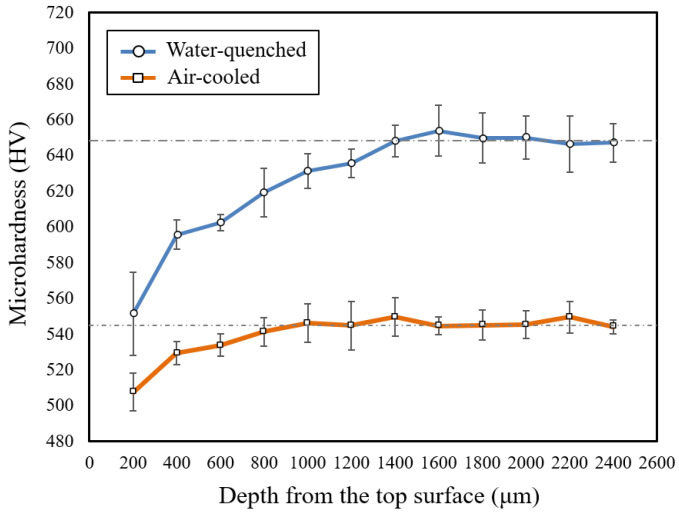
Vickers microhardness profile of the furnace-heated sample (water-quenched and air-cooled) showing a decrease in surface microhardness from the top surface.

Figure 10 shows the Vickers microhardness results of the furnace-heated samples. As seen from the results, the microhardness of both samples has increased significantly after the furnace heat treatment. This is due to the carbide dissolution during the heat treatment, which increases the carbon supersaturation and lattice residual stress of martensite. Comparing the two furnace-heated samples, the water-quenched sample was found to have a higher microhardness compared to the air-cooled sample. The bulk hardness of the water-quenched sample is about 650 HV, whereas the air-cooled sample’s bulk hardness is about 545 HV. This variation is due to the faster cooling rate of the water-quenched sample. The cooling rates in water and air are approximately 200–400 °C/s and 3–5 °C/s respectively [21]. However, comparing it to the previous laser surface hardening result, the achieved hardness of laser surface hardened sample is still slightly higher than the water-quenched furnace-heated sample. This could be due to several reasons. Firstly, the temperature during laser surface hardening is much higher than furnace heating of 980 °C, which results in complete or almost complete dissolution of carbides. Carbon dissolution in austenite is higher at higher austenizing temperatures. Secondly, the cooling rate in a laser surface treatment is much faster than the cooling rate in water quenching [22].

The microhardness results of both samples in Figure 10 show that decarburization has occurred in both samples down to the depth of about 1400 μm for the water-quenched sample and 1000 μm for the air-cooled sample. This decarburization depth difference is not as expected. Intuitively, the air-cooled sample should have a deeper decarburization depth since it was exposed to the oxidation condition at a longer time (slower cooling rate). This discrepancy could be due to a few reasons. Firstly, the high fluctuation of hardness may have caused inaccurate results in calculating the average microhardness of each location of interest, thus affecting the depth estimation. This can be seen from the large standard deviation range in the graph. This deviation may shift the depth estimation by a few hundred microns. Secondly, for the air-cooled sample, the slow cooling process may not only influence the decarburization process, but also may induce other microstructural changes such as grain growth. The microstructural changes during the slow cooling process may influence the hardness profile of the sample, such that the decarburization determination is worse. Therefore, the water-quenched sample is more suitable for the decarburization comparison to the laser-hardened sample. Comparing the hardness results, the hardness reduction in the furnace-heated sample is higher than the laser-hardened sample. For the water-quenched sample, the hardness drop is about 15%.

**Figure 11:**
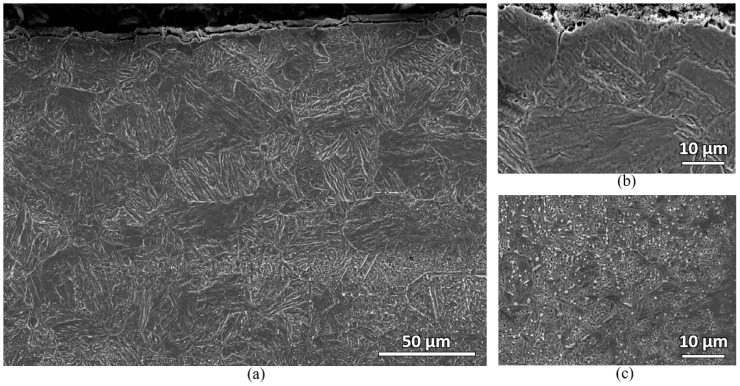
Secondary electron micrographs of the water-quenched furnace-heated sample showing martensitic microstructure with almost no carbide remaining at the near top surface of the sample: (**a**) low magnification near the top surface; (**b**) high magnification near the top surface; (**c**) high magnification at the bulk carbon location of the sample.

Microstructural characterizations of the furnace samples were also carried out. Both water-quenched and air-cooled samples show similar microstructures. Figure 11 shows the secondary electron micrographs of the water-quenched sample. Two distinct microstructures can be observed near the top surface and at the bulk carbon location of the sample. At the near top surface, large prior austenite grain (PAG) containing martensitic microstructure with almost no carbide is observed. However, at the deeper bulk carbon location, martensitic microstructure with numerous carbides is observed. In Figure 11a, carbide banding can also be observed. This indicates that there might be an anisotropic behavior in the material. The fluctuation of hardness in Figure 10 may be caused by this anisotropic property of the sample. However, this fluctuation in hardness does not change the significant surface hardness drop observations of the samples (see Figure 10).

**Figure 12:**
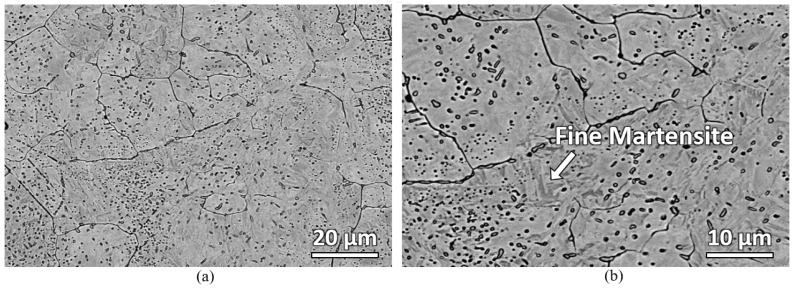
Backscattered electron micrographs of the water-quenched furnace-heated sample at bulk carbon location showing carbides distributed in martensitic matrix: (**a**) lower magnification and (**b**) higher magnification.

A deeper look at the bulk carbon location of the furnace sample was carried out using backscattered electron microscope. Figure 12 shows the backscattered electron micrograph of the bulk carbon location of the water-quenched furnace-heated sample. Fine martensite with numerous carbides is observed.

**Figure 13:**
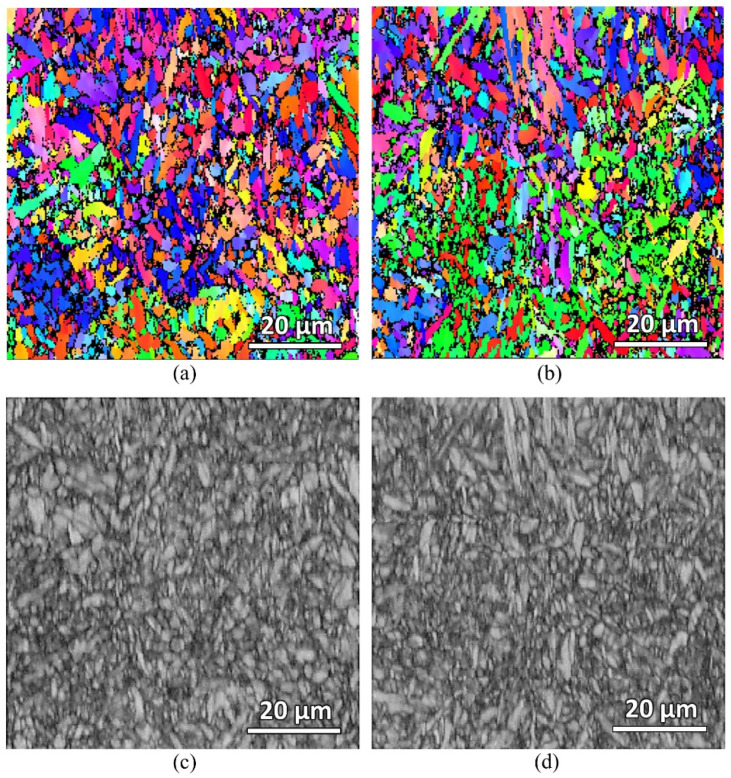
EBSD IPF-Y and band contrast maps of the water-quenched (**a**,**c**) and air-cooled (**b**,**d**) furnace heat-treated samples.

EBSD scanning of the furnace-heated samples were also carried out to check on the martensitic grain size of the samples. Figure 13 shows the EBSD IPF-Y and band contrast maps of both the water-quenched and air-cooled samples. The average martensitic grain size of both samples was measured to be the same, about 1.8 μm. This measurement was performed using the EBSD post-processing software based on the indexed grains. However, due to the very fine martensitic grains of the samples (as seen from the band contrast maps), the indexed grains are not accurately captured. Significant unindexed regions can be seen in the IPF-Y maps, which were due to the insufficient resolution of step size. More advanced equipment with a better step size resolution is needed to accurately quantity and compare the grain sizes.

### 3.3. Modelling

**Figure 14:**
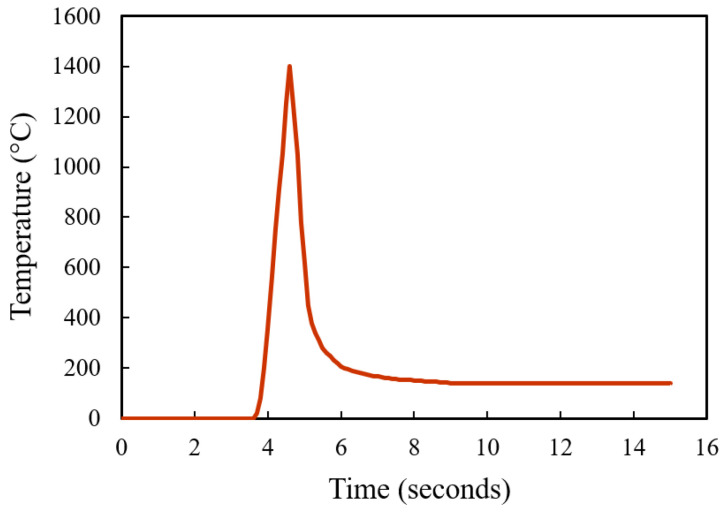
A typical laser thermal profile used as the laser surface hardening temperature input in simulation.

To further verify the decarburization results, simulations on the carbon content change under the heat treatment processes were carried out. A simple modelling of AISI 420SS for decarburization simulation was carried out using a diffusion module (DICTRA) package in ThermoCalc. A simple one-dimensional model was used. The carbon concentration profile along the depth is simulated. At the start, a single-phase austenite region containing the C and Cr weight percentage nominal composition of the AISI 420SS was used. Both furnace-heated hardening and laser surface hardening were modelled. For the furnace heat treatment, the parameter of simulation matches the parameters used in the experiment, 980 °C for 2 h on a 10 mm thickness sample. Similarly, for the laser surface hardening, a typical thermal profile was used as the simulation thermal input (see Figure 14).

During the heat treatment, decarburization can occur when carbon diffuses out from the steel surface under a particular atmospheric condition. The basic chemical reaction occurring during a decarburization is:

(1) $$C+{\;\mathrm{CO}}_{2}{}_{\left(g\right)}\;↔\;2{\mathrm{CO}}_{\left(g\right)}$$

This reaction is reversible. When the partial pressure of CO exceeds the partial pressure required to maintain a given carbon content, surface carburization occurs where the reaction goes from right to left. Reversely, when the CO_2_ partial pressure is higher than the CO content, the reaction will go from left to right, resulting in decarburization. Knowing the partial pressure of CO_2_ and CO during the heating process, together with the material initial composition and heating temperature, the equilibrium weight percentage of C in the steel surface during the heat treatment can be calculated using the following equation [9,23]:

(2) $$\mathrm{wt}\%\;C=\frac{1}{Kf_{c}}\frac{P_{\mathrm{CO}}{}^{2}}{P_{{\mathrm{CO}}_{2}}}\;$$

where $P_{\mathrm{CO}}$ and $P_{{\mathrm{CO}}_{2}}$ are the partial pressure of CO and CO_2,_ respectively, *K* is the equilibrium constant, and *f_c_* is the activity coefficient of carbon. This equilibrium constant is based on the fundamental law of mass action, for the reaction in Equation (1) whereby the relationship between the gaseous components and the carbon in solution of austenite is described. Equilibrium constant *K* is a function of temperature and can be obtained using Equation (3). T is the temperature in Kelvin. For the activity coefficient of carbon, it can be calculated using the Equation (4). T is also temperature in Kelvin.

(3) $$\log K=-\frac{8918}{T}+9.1148\;$$

(4) $$\log\;f_{c}=\frac{2300}{T}-2.24+\frac{179+8.9\mathrm{wt}\%\mathrm{Si}}{T}\mathrm{wt}\%C+\left(\frac{62.5}{T}+0.041\right)\mathrm{wt}\%\mathrm{Si}\;$$

Apart from the reaction of carbon with oxygen as shown in Equation (1), carbon also reacts with water vapor and iron oxides during the heat treatment. However, for simplification, these reactions were not considered in the analysis. Only the reaction in Equation (1) was considered and the equilibrium carbon concentration calculated from Equation (2) was used as the boundary condition during the heat treatment in the simulation.

During the heat treatment, carbon also diffuses from the interior to the surface of the steel. This diffusion of carbon inside austenite can be determined based on the Fick’s second law of diffusion, and it was solved numerically using ThermoCalc DICTRA software with a method developed by Andersoon and Ågren [24]. The carbon concentration profile along the sample depth was produced from the simulation. The carbon loss near the top surface provides a rough indication on the decarburization depth of the heat treatment process.

**Figure 15:**
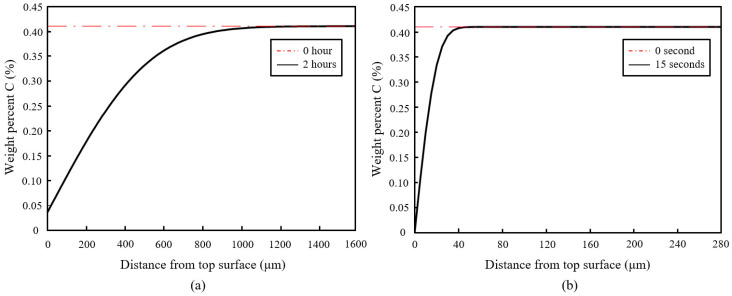
Simulation results showing the estimated decarburization depth of about: (**a**) 1200 μm for furnace-heated sample and (**b**) 40 μm for laser surface-hardening sample.

Figure 15 shows the simulation results of both furnace and laser surface hardening treatments. The temperature–time diagram in Figure 14 was used as the thermal input for the laser surface hardening prediction. For the furnace-hardening prediction, the same heating parameter used in the actual experiment, 980 °C for 2 h, was used as the prediction input. Results show that for the furnace heat treatment, the decarburization depth was estimated to be about 1200 μm, whereas it was 40 μm for the laser surface-hardening process. These simulation results are in good agreement with the experimental results (see Figure 9 and Figure 10). From the experiment microhardness result, the water-quenched furnace-heated sample has a decarburization depth of about 1400 μm, whereas for air-cooled, the sample has the decarburization depth of about 1000 μm. For the laser-hardened sample, the decarburization depth was measured to be about 40 μm from the nano hardness result. However, it is to be noted that the quantitative value obtained from this simulation might not be a perfectly accurate prediction of the real process. It should only serve as an estimation. This simulation used a typical laser thermal profile as the input and only considered the C and Cr element in the model, hence the effects of other alloying elements were not captured in simulation. Cr was considered as it is the major alloying element, whereas C is the element of interest for this decarburization study.

Decarburization was observed to have occurred in both furnace heating and laser surface hardening of AISI 420 martensitic stainless steel. In furnace heating (water-quenched), the bulk material of AISI 420SS has successfully been hardened to about 650 HV with 15% drop in hardness near the top surface due to decarburization. The decarburization depth is about 1400 μm. As for laser surface hardening, 200 μm region near the top surface of the steel has successfully been hardened to about 675 HV, and a slight 3% drop in hardness with about 40 μm decarburization depth was observed. Decarburization does occur in laser surface hardening of AISI 420SS; however, it might not be substantial to offset its effectiveness in hardening steel’s surfaces. Comparing to conventional air furnace heat treatment, laser surface hardening is faster and results in less decarburization. However, it is only useful when only a small top region near the steel’s top surface needs to be hardened. For a part where the whole bulk material needs to be hardened, the vacuum/high pressure gas quenching furnace method can be considered.

Comparing the results to the literature, currently, there no work was found to have specifically investigated the decarburization of AISI 420SS laser surface-hardening process. Several studies have worked on the laser surface hardening of AISI 420SS [25,26,27,28], however no focus was directed on the carbon loss near the top surface. This study hopes to fill this gap by systematically observing the phenomena near the top surface with the help of nano indentation. Two experiments were found to have used relatively finer microhardness measurement intervals along the sample depth. In the experiment conducted by Netprasert et al. [25], by observing the provided microhardness profile result, no decarburization was observed. This may be due to the extremely low pulsed laser power (15 and 21 W) used. Although the increase of surface hardness was found to be comparable to our result, about three times of the original, the hardened depth was found to be very shallow, only about 40 μm. This smaller hardened depth may not be sufficient for some surface-hardening needs. In the experiment carried out by Zirehpour et al. [26], no decarburization was observed in the microhardness profile result either. This could be attributed to two reasons. Firstly, they used a lower-pulsed laser power (300 and 350 W) and a higher scanning speed (30 and 35 mm/s) for the laser surface-hardening process. A low laser power and high scanning speed would have resulted in limited heat input to the substrate, which can also be observed from the smaller hardened layer produced. Secondly, they provided surface protection to the material by blowing argon gas to the material surface during the laser hardening process. These shows that decarburization would only occur in the laser surface hardening of AISI 420SS when a relatively high heat energy input was used. High heat energy input will result in a deeper hardened layer; however, decarburization could occur. Low heat energy input will be decarburization-free, but the hardened layer depth is small. Alternatively, decarburization can also be avoided if a surface protection, such as blowing argon gas to the material’s surface, can be provided during the laser surface-hardening process.

## 4. Conclusions

In this study, surface decarburization of AISI 420 martensitic stainless steel during the laser surface-hardening process was investigated. Results showed that even though laser surface hardening is fast, decarburization can still occur if high heat input is used. However, the decarburization intensity and depth are not as substantial as in the air furnace heat treatment. In a laser-hardened sample, surface hardness was increased by more than three times, from 200 HV to 675 HV, and the decrease in hardness due to decarburization was estimated to be about 3% with the decarburization depth of about 40 μm. As for the air furnace heat-treated sample, the sample’s bulk hardness was increased to 650 HV and a much higher decrease in hardness (of about 15%) with decarburization depth of about 1400 μm was observed on the sample’s surfaces. Laser surface hardening can generate a higher hardness with negligible decarburization as compared to air furnace heat treatment. This suggests that laser surface hardening is still a useful method for surface- or localized-hardening processes.

## Figures and Tables

**Table 1:** Chemical composition (wt.%) of AISI 420 martensitic stainless steel.

C	Si	Mn	Cr	Ni	Cu	Al	Mo	V	P	S	Fe
0.41	0.33	0.74	12.69	0.16	0.05	0.017	0.05	0.046	0.025	0.001	Bal.

## Data Availability

Not applicable.
